# Dr. Bristowe on the Registration of Nurses

**Published:** 1888-06-09

**Authors:** 


### Dr. Bristowe on the Registration of Nurses.

The annual meeting of The Hospitals Association was held at the Westminster Town Hall on May 30th, the President, Dr. J. L. Bristowe, in the chair. There were present among others Lieut.-General Keatinge, Mr. H. C. Burdett, Mr. Timothy Holmes, Mr. F. D. Mocatta, Surgeon-Major Ince, J. A. Steele, etc.

The President said—Ladies and Gentlemen,—It is my duty to submit the first resolution, namely, "That the report of the Council be adopted and entered on the minutes, and that it be printed and circulated under the direction of the Council." But I trust that, before I put this resolution to the vote, I may be allowed to make a longer speech than I think it has been customary for the chairman to make at our annual meetings, for the year has in some respects been an eventful year for the Association, and there are topics only touched upon in the report which demand, I think, further public treatment from our point of view than they have yet received.

Two matters of considerable interest have engaged the attention of the Association during the past year; about which a good deal of misconception appears to prevail in some quarters, and which have been made the subject of much acrimonious criticism by certain people; and to which, mainly for these reasons, I think it right to give special consideration. I refer to the registration of nurses and the National Pension Fund for Nurses.

The subject of the registration of nurses originated in The Hospitals Association. Early in 1886 a sectional committee was appointed on "nursing and the domestic management of hospitals;" and, in accordance with the principle on which the Association always acts, namely, that committees on special subjects should consist of those who have specia· knowledge of those subjects or take a special interest in them, this committee was composed exclusively of the matrons or lady-superintendents of hospitals. The object of the committee was "to secure co-operation or intercourse for mutual assistance and information between the ladies engaged in nursing throughout the country;" and one of the ladies most active in its formation explained at the first meeting that the reason which actuated her "was the need she had herself experienced of mutual co-operation in connexion with the responsible duties which lady-superintendents and matrons have to discharge at the various hospitals." Very shortly after the formation of the committee the question of the registration of nurses was brought before it by Mrs. Bluett, and a sub-committee was appointed "to consider the advisability of adopting a uniform nurse's certificate, &c." The sub-committee prepared a scheme of registration, which in a slightly modified form was finally adopted by the sectional committee, and was referred by it to the Council of the Association. A meeting of the Council was then called, to which the members of the sectional committee on nursing were invited; at which meeting the scheme prepared by the sectional committee was discussed, and, with one alteration, unanimously adopted. That alteration consisted in reducing the term of service to be required of nurses admitted to the register; and I have no doubt that if any scheme of registration were to come into general operation the reduction made by the Council, or even a larger reduction, would be found absolutely necessary.

It is a curious, and, I believe, generally recognised fact, that committees of ladies (however excellent and charming these may be individually) are unbusinesslike and capricious. And it is not altogether surprising, perhaps, that the Sectional Committee broke up towards the end of 1887, within two years from its formation, partly from internal causes, partly because some of its members took offence at the action of the Council in regard to the registration scheme; the sole assigned ground of offence being that the Council had modified the scheme as sent up to them by the committee in the single detail above specifie l, a modification which nevertheless had been accepted without protest by their representatives at the time at which it was made.

I may state that although the committee resigned, by no means all its members quarrelled with the Council, and that subsequently to this resignation (namely, on December 5th) three of the offended ladies met in committee, and passed the following resolution: "The members of the Sectional Committee, feeling that they have been made responsible for a scheme of registration of which they do not approve (viz., the one year's training) . . . feel called upon to resign their connexion as the committee. These now present declare that they are not able to do any good for the nursing profession under such irregular and unbusinesslike conditions. They wish to record their decided opinion that the registration of nurses should be the work of a legally-constituted body."

At a meeting of the Association, held on December 14th, at Guy's Hospital, under the chairmanship of Mr. Lushington, the treasurer of that institution, Dr. Steele read a paper, which had been announced at the beginning of the session, upon this subject of the registration of nurses. At this meeting, the former committee having ceased to exist, a new committee, composed partly of matrons of hospitals and partly of gentlemen, was appointed.

This committee, having elected Dr. Steele chairman proceeded at once to do what no doubt should have been done in the first instance by the previous committee, namely, to ascertain the opinions with regard to the registration of nurses entertained by the establishments in England and Scotland which profess to educate nurses. The result, if not disappointing, was certainly not what had been anticipated, for the committee found that the majority of them, including all the leading and best-known training schools, desired to be let alone, on the grounds that they had all established registers of their own to meet their special requirements; that they should lose rather than gain by any system partaking of amalgamation; and that the time had not arrived for a central organisation to be delegated with authority to control the action of the various bodies, commercial and charitable, entrusted with the management of nurses.

The report which the committee drew up on the subject concluded as follows: "The results of the inquiry all tend to confirm the opinion that it would be premature on the part of the committee to recommend to the Council a common basis cn which a general system of registration for nurses should be framed, however advisable it may be for every hospital, being also a nurse-training institution, to amend and consolidate its own educational training in keeping with the improvements which of late years are well known to have been introduced into most establishments of this character."

This report, which ought to be read and well thought over by all persons who are interested in the question of the registration of nurses, was adopted by the Council on the 28th of last March.

There, for the present, all connexion between The Hospitals Association and the registration of nurses ceases. The Association entered upon the subject in good faith, and with the honest belief which many persons still entertain that a scheme of registration was feasible and would be accepted. It has been reluctantly forced to conclude that such a scheme is, at any rate for the present, neither feasible nor acceptable.

The subject of the registration of nurses is one to which I have paid no special attention, and upon which I hold no strong opinions. But I, for one, shall certainly take no part in forcing anything of the kind on the great nurse-training schools of this country. It is to these schools that we owe the marvellous development of nursing, and the great improvement in the position and quality of nurses, which the last twenty or thirty years have witnessed; it is to these mainly that we must look in the immediate future for still further advances in the same direction; and it is they who are most qualified to say whether a general scheme of registration would be beneficial or not, and to act, if need be, as pioneers in such a movement.

I have not referred to the later proceedings of those ladies who seceded from The Hospitals Association, and under high auspices have started a registration scheme for nurses on their own account, for they do not immediately concern us. My object has been to explain the relation of The Hospitals Association to the registration scheme, and to show that from first to last it has not been influenced by temper or any unworthy motive.

[The conclusion of Dr. Bristowe's address will be found in "The Nursing Mirror."]

